# Levels of support for the licensing of tobacco retailers in Australia: findings from the National Drug Strategy Household Survey 2004-2016

**DOI:** 10.1186/s12889-020-08920-1

**Published:** 2020-05-24

**Authors:** John Baker, Mohd Masood, Muhammad Aziz Rahman, Stephen Begg

**Affiliations:** 1 Rural Department of Community Health, La Trobe Rural Health School, La Trobe University, P.O Box 199, Bendigo, VIC 3552 Australia; 2 Department of Dentistry and Oral Health, La Trobe Rural Health School, La Trobe University, Bendigo, VIC Australia; 3 School of Nursing and Healthcare Professions, Federation University Australia, Berwick, VIC Australia; 4 School of Nursing and Midwifery, La Trobe University, Melbourne, VIC Australia

**Keywords:** Tobacco, Public opinion, Licensing, National drug strategy household survey, Retail, Tobacco policy

## Abstract

**Background:**

Assessing public opinion towards tobacco policies is important, particularly when determining the possible direction of future public health policies. The aim of this study was to describe the implementation of tobacco retailer licensing systems by state and territory governments in Australia, and to use the National Drug Strategy Household Survey (NDSHS) to assess levels of public support for a retailer licensing system in each jurisdiction over time and by a range of socio-demographic and behavioural attributes.

**Methods:**

National and state/territory estimates of public support for a tobacco retailer licensing system were derived as proportions using NDSHS data from 2004 to 2016. The effect of one’s jurisdiction of residence on the likelihood of supporting such an initiative in 2016 was assessed using logistic regression while controlling for various socio-demographic and behavioural characteristics.

**Results:**

Public support for a tobacco retailer licensing system ranged from a high of 67.2% (95% CI 66.5–67.9%) nationally in 2007 and declined to 59.5% (95% CI 58.9–60.2%) in 2016. In 2016, support was greatest amongst those from Tasmania, those aged 50 years and older, females, those from the least disadvantaged areas, those living in major cities, never-smokers and never-drinkers. After adjusting for the socio-demographic and behavioural attributes of respondents, those from Queensland were significantly less likely to support a licensing system (adjusted OR = 0.85, 95% CI 0.77–0.94) compared to those from other jurisdictions, while those from Tasmania were significantly more likely to support a licensing system compared to those from other jurisdictions (adjusted OR = 1.29, 95% CI 1.09–1.52).

**Conclusions:**

A clear majority of the public support a tobacco retailer licensing system, regardless of whether or not such a system is already in place in their jurisdiction of residence. Tobacco control initiatives other than a retailer licensing system may explain some of the residual variations in support observed between jurisdictions.

## Background

Despite Australia being at the forefront of tobacco control initiatives globally since the 1970s, recent data [1] suggests that declines in national daily smoking rates are starting to slow, and further gains may be increasingly hard to achieve. Meanwhile, smoking is still a leading cause of preventable mortality, with 20933 or 13.3% of deaths in 2015 caused by tobacco use [1]. Tobacco also remains responsible for 9.0% of the total burden of disease in Australia due its role in causing a range of chronic diseases, including heart disease, stroke, cancer, diabetes, emphysema and renal disease [1].

Contributing to the persistence of smoking as a public health problem is the fact that tobacco products are sold alongside many other everyday consumer items by an estimated 29907 (2014) to 40000 (2014) tobacco retailers across Australia [2–5]. Research in New South Wales (NSW), for example, indicated that there were five times as many tobacco retailers compared to pharmacies, and eight times as many tobacco retailers as there are Australia Post outlets in 2012 [6].

Licensing or registration is mandatory across all jurisdictions of Australia for a number of occupations where public health or safety is a concern, including electricians, civil engineers, dentists, pharmacists, doctors, alcohol retailers, food handlers and gaming operators, however, this is not the case for tobacco retailers [7, 8]. Several researchers have suggested that licensing tobacco retailers can have a beneficial impact on population health when used to regulate the number of retailers, to prevent underage sales, to improve compliance with existing legislation, and as a means to reduce density and proximity in lower socioeconomic (SES) neighbourhoods and near schools [9, 10].

A number of jurisdictions globally have implemented licensing systems for tobacco retailers, including Singapore, Hungary, France, Finland, New York State, California and San Francisco [11–15]. San Francisco has limited the number of retailer licenses to 45 per suburb, and the sale of tobacco within approximately 150 m (500 ft) of a school or another retailer is prohibited [15, 16]. In 2013, Hungary implemented legislation to only allow the sale of tobacco from Government-licensed or designated retailers (called National Tobacco Shops) to reduce youth smoking. It was anticipated that the number of retailers legally allowed to sell tobacco would reduce from 42000 to approximately 7000 retailers [14].

In Australia, the regulation of tobacco retailers is a state and territory government responsibility, with six out of the eight state and territory jurisdictions having adopted a licensing or registration system for this sector to date [17]. However, the literature about these systems and public attitudes towards them is surprisingly limited. The National Drug Strategy Household Survey (NDSHS) regularly collects data on the personal use of licit and illicit drugs, as well as attitudes and perceptions of drug use and policy in the community [18, 19]. Although this survey has been used to assess attitudes towards alcohol policies previously, to date it has not been used to assess attitudes towards tobacco-related policies [20–22]. The aim of this study was therefore twofold: 1) to describe the implementation of tobacco retailer licensing systems by state and territory governments in Australia, and 2) to use the NDSHS to measure levels of public support for a retailer licensing system in each jurisdiction in Australia over time and by a range of socio-demographic and behavioural characteristics.

## Methods

### Implementation of licensing for tobacco retailers

Relevant legislation was reviewed to determine the implementation year and annual cost (in Australian Dollars, $A) for existing tobacco retailer licensing systems in each state and territory jurisdiction. This information was supplemented by a submission to the recent Queensland (QLD) Government Inquiry into Tobacco Licensing Arrangements [17].

### Public support for licensing of tobacco retailers

The NDSHS is a triennial nationally representative survey of those aged 12 years and older about issues relating to alcohol and drug use in Australia [18]. Participants are selected through stratified, multistage random sampling of households, with a response rate for the most recent survey of 51.1% [23]. For the 2004, 2007 and 2010 surveys, respondents aged 14 years and older were asked the question, “Thinking now about the problems associated with tobacco use, to what extent would you support or oppose measures such as implementing a licensing scheme for tobacco retailers?” This question was modified in the 2013 and 2016 surveys to “…to what extent would you support or oppose measures such as implementing a *national* licensing scheme for tobacco retailers?” (emphasis added). Response options ranged from “Strongly oppose” to “Strongly support” and included “Don’t know enough to say” [19].

National and state/territory estimates of public support for a tobacco retailer licensing system between 2004 and 2016 were derived as proportions (“strongly support” and “support” over all responses) using the recommended weighting technique [18]. Ninety-five percent Confidence Intervals (CIs) were calculated using $1.96\times \surd \left(\left(\hat{p}\left(1-\hat{p}\ \right)\right)/n\right)$, where $\hat{p}$ was the weighted sample proportion and *n* was the unweighted sample size. The effect of one’s jurisdiction of residence on the likelihood of supporting such an initiative (“strongly support” and “support” versus “strongly oppose”, “oppose” and “neither support nor oppose”) were assessed using logistic regression analysis in SPSS. The following socio-demographic and behavioural attributes were included as possible confounders: age (14–17 years, 18–29 years, 30–49 years, 50 years and over), sex, SES of area of residence (measured in quintiles from most disadvantaged to least disadvantaged using Australian Bureau of Statistics Socio-Economic Indices for Areas [SEIFA] scores), remoteness of area of residence (major cities, inner regional, regional/remote/very remote), smoking status (daily smoker, current occasional smoker, ex-smoker, never-smoker) and alcohol use (daily drinker, weekly drinker, less than weekly drinker, ex-drinker for greater than 12 months, never-drinker). Due to differences in the way in which these attributes are recorded in the NDSHS over time, logistic regression analysis was only attempted using a subsample from 2016. The subsample was summarised in terms of unweighted numbers and proportions for each of the attributes, with differences between supporters and others being assessed using chi-square tests.

## Results

### Implementation of licensing for tobacco retailers

Details about existing tobacco retailer licensing systems in Australia are summarised in Table 1. All Australian states and territories required tobacco wholesalers and retailers to pay a fee based on the percentage of the value of tobacco products sold until this regulatory approach was declared constitutionally invalid in 1997. South Australia (SA) was the first jurisdiction to reintroduce a constitutionally valid licensing system in 1998, followed by the Australian Capital Territory (ACT) and Tasmania (TAS) in 2000, the Northern Territory (NT) in 2003, Western Australia (WA) in 2007 and NSW in 2009. Five of the eight jurisdictions (ACT, SA, NT, WA and TAS) have a “positive” licensing system where tobacco retailers are required to apply for registration and pay an annual fee, ranging from $A242.00 (2019–20) in the NT to $A1161.54 in TAS (2019). TAS also requires personal vaporiser retailers (e.g. sellers of electronic cigarettes) to register and pay an annual fee of $A583.20. WA requires indirect sellers, where the seller and the customer are not in the same location (e.g. sale by fax, telephone or mail order, or via the internet), to also apply for a licence. Two jurisdictions require wholesalers to apply for registration and to pay an annual fee, ranging from $A360.00 in the ACT (2016) to $A715.00 in WA (2019). NSW currently has a “negative” licensing system whereby retailers are simply required to notify the government on a one-off basis if they sell tobacco. No annual fee is payable. Victoria (VIC) and QLD do not have any type of retailer licensing system in place. The QLD Government held an inquiry into tobacco retailer licensing in 2016, but legislation has not been introduced at this stage [17].

**Table 1:** Tobacco retailer licensing system by State and Territory in Australia

State/Territory	Licensing system type	Annual cost of licence ($A)	Implementation year
South Australia (SA)	Positive	$A297.00 (2019) [24]	1998 [25]
Australian Capital Territory (ACT)	Positive	Retail: $A540.00 (2017–18) [26] Wholesale: $A360.00 (2016) [17]	2000 [27]
Tasmania (TAS)	Positive	Tobacco products only: $A1161.54^a^ (2019) Tobacco products and personal vaporiser products^a^: $A1161.54 (2019) Personal vaporiser products^a^ only: $A583.20 (2019) [28]	2000 [29]
Northern Territory (NT)	Positive	$A242.00 (2019–20) [30]	2003 [31]
Western Australia (WA)	Positive	Retail: $A286.00 Indirect: $A289.00 Wholesale: $A715.00 (2019) [32]	2007 [33]
New South Wales (NSW)	Negative	No fee	2009 [34]
Queensland (QLD)	No licensing system	Not applicable	Not applicable [17]
Victoria (VIC)	No licensing system	Not applicable	Not applicable [35]

^a^Personal vaporiser products include electronic cigarettes (E-cigarettes). Note: Currency is in Australian Dollars ($A)

### Support for licensing of tobacco retailers

Public support for a tobacco retailer licensing system has remained above 50% since 2004, when the question was first asked in the NDSHS, ranging from a high of 67.2% (95% CI 66.5–67.9%) nationally in 2007 and declining to 59.5% (95% CI 58.9–60.2%) in 2016 (Additional file 1: Table S1). National and state/territory estimates of support for a tobacco retailer licensing system between 2004 and 2016). The highest level of support in a jurisdiction was 70.2% (95% CI 67.3–73.1%) for TAS in 2004; the lowest was 56.1% (95% CI 53.0–59.2%) for the NT in 2013, the year the new wording of the question was introduced. Support in the two jurisdictions currently without a tobacco retailer licensing system ranged from a high of 68.2% (95% CI 66.7–69.7%) in 2007 and declining to 60.9% (95% CI 59.6–62.2%) in 2016 for VIC, and from a high of 66.5% (95% CI 65.0–68.1%) in 2007 declining to 56.4% (95% CI 54.8–58.0%) in 2016 for QLD.

Table 2 summarises the unweighted subsample used in the logistic regression analysis. The distribution of levels of support for a tobacco retailer licensing system in 2016 were found to be different across categories of jurisdiction of residence, age, sex, smoking status and alcohol consumption but not SEIFA quintile or remoteness. In this subsample, support was greatest amongst those from TAS (69.1%), those aged 50 years and older (68.5%), females (68.9%), those from the least disadvantaged areas (70.6%), those living in major cities (68.1%), never-smokers (75.3%), and never-drinkers. Conversely, support was lowest amongst those from the NT (60.7%), those aged between 18 and 29 years (63.0%), males (64.1%), those from the most disadvantaged areas (63.2%), those living in outer regional, remote or very remote areas (61.2%), daily smokers (32.5%), and daily drinkers (57.9%). Support was below 50% only amongst daily and current occasional smokers.

**Table 2:** Socio-demographic and behavioural attributes of NDSHS respondents in 2016 and support for the implementation of a tobacco retailer licensing system

	Supporters (***N*** = 13431)	%	Others^**a**^ (***N*** = 6712)	%	***P*** value^**b**^
**Jurisdiction of residence**					
NSW	3564	67.9	1684	32.1	< 0.001
VIC	3243	68.3	1508	31.7	
QLD	2024	62.7	1206	37.3	
WA	1529	67.1	749	32.9	
SA	1239	66.4	626	33.6	
TAS	647	69.1	290	30.9	
ACT	608	68.8	276	31.2	
NT	577	60.7	373	39.3	
**Age (years)**					
14–17	405	66.1	208	33.9	< 0.001
18–29	1661	63.0	976	37.0	
30–49	4323	65.4	2290	34.6	
50 years and over	7042	68.5	3238	31.5	
**Sex**					
Female	7479	68.9	3382	31.1	< 0.001
Male	5952	64.1	3330	35.9	
**SEIFA Quintile**					
1 (Most disadvantaged)	2380	63.2	1388	36.8	0.085
2	2663	65.6	1398	34.4	
3	2624	66.2	1339	33.8	
4	2827	67.5	1363	32.5	
5 (Least disadvantaged)	2937	70.6	1224	29.4	
**Remoteness**					
Major Cities	9046	68.1	4245	31.9	0.017
Inner regional	2553	66.2	1305	33.8	
Outer regional/Remote/Very remote	1832	61.2	1162	38.8	
**Smoking status**					
Daily smoker	807	32.5	1673	67.5	< 0.001
Current occasional smoker	214	40.5	314	59.5	
Ex-smoker	3663	66.4	1854	33.6	
Never-smoker	8747	75.3	2871	24.7	
**Alcohol consumption**					
Daily drinker	820	57.9	597	42.1	< 0.001
Weekly	4970	63.5	2857	36.5	
Less than weekly	4774	67.8	2267	32.2	
Ex-drinker (> 12 months)	1263	69.7	550	30.3	
Never-drinker (full glass)	1604	78.4	441	21.6	

Note: Numbers are unweighted. SEIFA: Socio-Economic Index for Areas. ^a^ Includes Oppose, Strongly Oppose and Neither Support nor Oppose. Excludes those who answered “Don’t know enough to say” (*n* = 2157). ^b^ Derived from Pearson’s chi-square tests for categorical variables

Without controlling for the different socio-demographic and behavioural attributes of respondents, those living in QLD (unadjusted OR = 0.79, 95% CI 0.72–0.86) and the NT (unadjusted OR = 0.73, 95% CI 0.63–0.84) were significantly less likely to support a licensing system than those living in other jurisdictions (Model 1, Table 3). However, once the different socio-demographic and behavioural attributes of respondents were taken into account (Model 2, Table 3), those from QLD were significantly less likely to support a licensing system (adjusted OR = 0.85, 95% CI 0.77–0.94) compared to those from other jurisdictions, while respondents from TAS were significantly more likely to support a licensing system compared to those from other jurisdictions (adjusted OR = 1.29, 95% CI 1.09–1.52).

**Table 3:** Support for the implementation of a tobacco retailer licensing system by socio-demographic and behavioural attributes of NDSHS respondents in 2016

	Odds Ratio (OR)	95% C.I.	***P*** value
***Model 1: Unadjusted ORs***			
**Jurisdiction of residence**			< 0.001
NSW	1.00		
VIC	1.01	(0.93–1.10)	0.710
QLD	0.79	(0.72–0.86)	< 0.001
WA	0.96	(0.86–1.07)	0.500
SA	0.93	(0.83–1.04)	0.242
TAS	1.05	(0.90–1.22)	0.491
ACT	1.04	(0.89–1.21)	0.609
NT	0.73	(0.63–0.84)	< 0.001
***Model 2: Adjusted ORs***			
**Jurisdiction of residence**			< 0.001
NSW	1.00		
VIC	1.04	(0.95–1.13)	0.393
QLD	0.85	(0.77–0.94)	0.002
WA	0.97	(0.87–1.09)	0.687
SA	0.92	(0.82–1.04)	0.223
TAS	1.29	(1.09–1.52)	0.003
ACT	0.98	(0.83–1.16)	0.856
NT	0.98	(0.81–1.18)	0.858
**Age (years)**			< 0.001
14–17	1.00		
18–29	1.50	(1.22–1.83)	< 0.001
30–49	1.88	(1.55–2.29)	< 0.001
50 years and over	2.10	(1.73–2.54)	< 0.001
**Sex**			
Female	1.00		
Male	0.89	(0.83–0.95)	< 0.001
**SEIFA Quintile**			0.085
1 (Most disadvantaged)	1.00		
2	1.06	(0.96–1.17)	0.251
3	1.05	(0.95–1.16)	0.295
4	1.09	(0.99–1.21)	0.072
5 (Least disadvantaged)	1.16	(1.04–1.29)	0.006
**Remoteness**			0.17
Major Cities	1.00		
Inner regional	0.97	(0.88–1.06)	0.509
Outer regional/Remote/Very remote	0.85	(0.76–0.95)	0.004
**Smoking status**			< 0.001
Daily smoker	1.00		
Current occasional smoker	1.50	(1.23–1.82)	< 0.001
Ex-smoker	3.94	(3.55–4.36)	< 0.001
Never-smoker	5.92	(5.37–6.53)	< 0.001
**Alcohol status**			< 0.001
Daily drinker	1.00		
Weekly	1.05	(0.92–1.19)	0.435
Less than weekly	1.25	(1.10–1.42)	0.001
Ex-drinker (> 12 months)	1.39	(1.19–1.62)	< 0.001
Never-drinker (full glass)	1.82	(1.54–2.16)	< 0.001

*SEIFA* Socio-Economic Index for Areas

## Discussion

This study has described the implementation of different tobacco retailer licensing systems by Australian state and territory governments in recent years, and has assessed levels of support for such policies over time and by various socio-demographic and behavioural attributes of respondents. The findings indicate that despite an inconsistent approach to this issue by governments, a clear majority of the public are supportive of a tobacco retailer licensing system, regardless of whether or not such a system is already in place in their jurisdiction of residence. While there is variation between jurisdictions in levels of support, this variation is attenuated in the case of QLD, which has no licensing system, or disappears altogether in the case of NT, which has a positive licensing system, when the different socio-demographic and behavioural attributes of the respective populations are taken into account. Levels of support in TAS on the other hand, which also has a positive licensing system, become significantly higher after controlling for these factors. Tobacco control initiatives by state and territory governments other than a retailer licensing system may explain some of these residual variations.

The study also revealed majority support for the licensing of tobacco retailers across nearly all categories of the socio-demographic and behavioural attributes analysed (with the exception of current smokers), with the highest likelihood of support amongst those respondents from TAS, those aged 50 years and over, females, those from high-SES areas [SEIFA], those living in major cities, never smokers and never drinkers. The patterns of support across these attributes reflected broader trends in the social determinants of health, with levels of support increasing with increasing urbanicity and SES advantage. Only about one-third of current smokers indicated support for a retailer licensing system, but this group only made up less than 15% of the population. These patterns are important to consider as Australian research suggests there is greater tobacco retailer density and higher smoking rates in lower-SES areas and regional and remote areas [36–40].

Our findings also indicate that around two-thirds of those aged 14–17 years were supportive of a tobacco retailer licensing system. The minimum purchasing age for tobacco products in Australia is 18 years, however recent research found that 9% of 12–15 year-old smokers purchased cigarettes themselves, and this figure increased to 24% amongst 16–17 year-old weekly smokers [41], suggesting that many retailers are not adhering to the relevant legislation. Chapman and Freeman [9] argue that tobacco retailer licensing should be implemented and heavily restricted, with a potential loss of licence for breaches of conditions. Retailer compliance with tobacco control laws such as preventing sales to minors, the sale of illicit tobacco, and the promotion of tobacco products could be improved through the implementation of a positive licensing system that generates a sustainable revenue cycle, as recommended by Quit Victoria [5]. An Australian study found a reduction in attempted tobacco purchases amongst minors when there was sustained and vigorous enforcement of underage sales legislation [42].

Without a comprehensive tobacco retailer licensing system in NSW, VIC and QLD, it is difficult to accurately determine both the number of retailers in these jurisdictions and how many are complying with the relevant legislation at any point in time. Quit Victoria has estimated that there were approximately 8000 retailers in VIC alone in 2014 [5]. Local governments throughout VIC are provided with funding to undertake regular compliance checks, education visits and test purchasing amongst tobacco retailers. However, these activities only target retailers known to local government, whereas research suggests that it is the others that are less likely to comply with relevant tobacco retailing laws [43]. Recent research in NSW, for example, identified one unlisted tobacco retailer for every 12.6 registered retailers, and those unlisted retailers were significantly more likely to breach in-store legislation [44]. Retailers in more disadvantaged areas were also more likely to breach in-store regulations than those operating in less disadvantaged areas. This suggests that a negative licensing system does not improve compliance with existing retail legislation, nor does it necessarily lead to the accurate identification of all tobacco retailers [44].

A number of best-practice solutions to reduce the density of tobacco retailers would be facilitated by the adoption of more consistent tobacco retailer licensing policies in Australia, including limiting the number of retailers within specified geographical areas, imposing minimum-distance requirements for retailers near schools, creating a maximum number of retailers proportional to population size, and limiting the types of businesses that can sell tobacco. Such initiatives could be adopted and enforced at the local level, in an approach that is similar to the one adopted to address alcohol availability in VIC [11, 45, 46]. It would seem, therefore, that the introduction of a comprehensive, positive tobacco retailer licensing system is the logical next step towards further strengthening tobacco control measures in NSW, VIC and QLD. Policy-makers in these jurisdictions should find encouragement in the high levels of public support for such policies and also by research in SA, which demonstrates that the number of tobacco retailer licences purchased or renewed can be reduced simply by increasing tobacco retailer licensing fees to as little as $A200.00 per annum [10].

A report commissioned by the Federal Government in 2002 identified difficulties in nominating a constitutional head of power to oversee responsibility for the implementation of a tobacco retailer licensing system at the national level [47]. Despite this, the report urged the Federal Government to legislate for a tobacco retailer licensing system that overrides all existing state and territory approaches, whilst emphasising the importance of setting a licensing fee at an acceptable rate to ensure that it is not simply a revenue-raising exercise [47]. Such an initiative by the Federal Government would be consistent with the World Health Organization’s (WHO) Framework Convention on Tobacco Control (FCTC), which promotes the implementation of *“…licensing, where appropriate, to control or regulate the production and distribution of tobacco products in order to prevent illicit trade.”* [48]

This study used data from the NDSHS, which is a nationally representative survey of the attitudes and behaviours of Australians in relation to drug use with a reasonable response rate. However, the NDSHS data collection methodologies have changed over time, which may explain some of the trends presented. In 2004 and 2007, for example, the personal interview methodology was removed, with only ‘Drop and Collect’ and ‘Computer-Assisted Telephone Interview’ (CATI) methods employed. For 2010 and 2013, data were collected using only the Drop and Collect methodology. For 2016, a multi-mode collection approach was used, with respondents completing the survey online, via telephone or by paper [18]. It is not clear how these different methodologies might influence responses to the question about the implementation of a tobacco retailer licensing system.

The findings are also limited by the way in which the NDSHS determines support for the implementation of a tobacco retailer licensing system: only one question was asked in relation to this hypothetical policy and no additional contextual information was provided. For example, respondents were not told whether a positive or negative licensing system was being proposed, whether retailers would be required to pay an annual registration fee, the cost of the fee, what that fee might be used for, or whether wholesalers would also be licensed. Many respondents from jurisdictions that already have a retailer licensing system in place may not be aware of this and their responses might change if this information had been provided.

Finally, there were also minor differences in the way the question was worded in the survey over time, with an emphasis on a ‘national’ licensing system from 2013 onwards. Again, it is not clear how these differences might influence responses before and after the change.

## Conclusions

The slowing decline in Australian smoking rates in recent years suggests the need for renewed investment in tobacco control activities [1, 49]. To further reduce smoking rates, Australian policy makers should consider reducing the availability of tobacco products through policies that have been shown to influence the density of tobacco retailers in communities [49]. This study has demonstrated consistent and widespread public support for the licensing of tobacco retailers while other studies have demonstrated the impact of such policies on tobacco product availability, particularly amongst minors. The uniform adoption of a comprehensive, positive licensing system for tobacco retailers across jurisdictions would seem to be a useful next step towards further strengthening tobacco control measures in Australia.

## Supplementary information

**Additional file 1: Table S1.** National and state/territory estimates of support for a tobacco retailer licensing system between 2004 and 2016.

## Data Availability

The data that support the findings of this study are available from the Australian Data Archive but restrictions apply to the availability of these data, which were used under license for the current study, and so are not publicly available. Data are however available from the authors upon reasonable request and with permission of the Australian Data Archive. Please contact the Australian Data Archive to request permission (ada@anu.edu.au).

## Abbreviations

ACT: Australian Capital Territory
CIs: Confidence Intervals
E-cigarette: Electronic cigarettes
FCTC: Framework Convention on Tobacco Control
NDSHS: National Drug Strategy Household Survey
NSW: New South Wales
NT: Northern Territory
QLD: Queensland
SEIFA: Socio-Economic Indices for Areas
SES: Socioeconomic Status
SA: South Australia
TAS: Tasmania
VIC: Victoria
WA: Western Australia
WHO: World Health Organization
