# Supplementary material for: Levels of support for the licensing of tobacco retailers in Australia: findings from the National Drug Strategy Household Survey 2004-2016
Source: BMC Public Health. 2020 May 24;20:773. doi: 10.1186/s12889-020-08920-1 (PMC7247215; doi:10.1186/s12889-020-08920-1)
Supplement: Supplementary file 1 — Additional file 1: Table S1. National and state/territory estimates of support for a tobacco retailer licensing system between 2004 and 2016. [file 12889_2020_8920_MOESM1_ESM.docx]

**Supplementary Table 1. National and state/territory estimates of support for a tobacco retailer licensing system between 2004 and 2016**

|  |  |  | **Year of survey** | | |  |  | |
| --- | --- | --- | --- | --- | --- | --- | --- | --- |
|  | **2004** | **2007** | **2010** | | | **2013** | **2016** | |
|  | **N, % (95% CIs)** | **N, % (95% CIs)** | **N, % (95% CIs)** | | | **N, % (95% CIs)** | **N, % (95% CIs)** | |
|  | |  |  | | |  |  | |
| **NSW** | 6240, 65.6 (64.5 – 66.8) | 5159, 66.6 (65.4 – 67.9) | 7224, 67.1 (66.0 – 68.2) | 6110, 63.3 (62.1 – 64.5) | | | 5795, 60.5 (59.2 – 61.8) | |
| **VIC** | 4804, 64.6 (63.2 – 65.9) | 3833, 68.2 (66.7 – 69.7) | 5257, 64.7 (63.4 – 66.0) | 4856, 62.3 (60.9 – 63.6) | | | 5279, 60.9 (59.6 – 62.2) | |
| **QLD** | 4626, 64.5 (63.2 – 65.9) | 3395, 66.5 (65.0 – 68.1) | 5203, 62.1 (60.8 – 63.4) | 4020, 58.9 (57.3 – 60.4) | | | 3573, 56.4 (54.8 – 58.0) | |
| **WA** | 2275, 63.0 (61.0 – 64.9) | 1863, 69.7 (67.7 – 71.8) | 2320, 63.7 (61.7 – 65.6) | 2378, 60.0 (58.1 – 62.0) | | | 2511, 59.9 (58.0 – 61.8) | |
| **SA** | 1845, 66.5 (64.3 – 68.6) | 1498, 64.9 (62.5 – 67.3) | 1929, 64.0 (61.8 – 66.1) | 1805, 58.8 (56.6 – 61.1) | | | 2105, 58.3 (56.2 – 60.4) | |
| **TAS** | 940, 70.2 (67.3 – 73.1) | 889, 68.6 (65.6 – 71.7) | 1002, 68.7 (65.8 – 71.5) | 1070, 62.8 (59.9 – 65.7) | | | 1037, 60.6 (57.6 – 63.6) | |
| **ACT** | 939, 66.2 (63.2 – 69.3) | 936, 66.7 (63.7 – 69.7) | 996, 69.4 (66.5 – 72.2) | 1018, 63.2 (60.2 – 66.1) | | | 982, 60.6 (57.6 – 63.7) | |
| **NT** | 839, 62.4 (59.1 – 65.7) | 776, 60.3 (56.8 – 63.7) | 980, 63.3 (60.2 – 66.3) | 977, 56.1 (53.0 – 59.2) | | | 1030, 56.8 (53.8 – 59.8) | |
| **Total** | 22508, 65.0 (64.4 – 65.7) | 18349, 67.2 (66.5 – 67.9) | 24911, 64.9 (64.3 – 65.5) | 22234, 61.4 (60.7 – 62.0) | | | 22312, 59.5 (58.9 – 60.2) | |
| Proportion indicating support (95% CI). | | |  | | |  |  | |
| ^a^ Weighted by absolute person weight (Weight 7). | | |  | |  | | |  |
